# Understanding Child Wasting in Ethiopia: Cross-sectional Analysis of 2019 Ethiopian Demographic and Health Survey Data Using Generalized Linear Latent and Mixed Models

**DOI:** 10.2196/39744

**Published:** 2023-02-08

**Authors:** Girma Gilano, Samuel Hailegebreal, Sewunet Sako, Firehiwot Haile, Kasarto Gilano, Binyam Tariku Seboka, Kefita Kashala

**Affiliations:** 1 Department of Health Informatics, School of Public Health College of Medicine and Health Sciences Arba Minch University Arba Minch, Southern Nation Nationalities Peoples' Region Ethiopia; 2 Department of Public Health, School of Public Health College of Medicine and Health Sciences Arba Minch University Arba Minch, Southern Nation Nationalities Peoples' Region Ethiopia; 3 Department of Public Health Arba Minch College of Health Sciences Arba Minch, Southern Nation Nationalities Peoples' Region Ethiopia; 4 Department of Health Informatics, School of Public Health College of Medicine and Health Sciences Dilla University Dilla, Southern Nation Nationalities Peoples' Region Ethiopia; 5 Department of Biomedical Science, School of Medicine College of Medicine and Health Sciences Arba Minch University Arba Minch, Southern Nation Nationalities Peoples' Region Ethiopia

**Keywords:** wasting, Generalized Linear Latent and Mixed Models, GLLAMM, under-five children, factors, Ethiopia

## Abstract

**Background:**

Wasting is an immediate, visible, and life-threatening form of undernutrition in children aged <5 years. Within a short time, wasting causes recurrent sickness, delayed physical and mental growth, impatience, poor feeding, and low body weight. The long-term consequences of wasting and undernutrition are stunting, inability to learn, poor health status, and poor work performance. Wasting remains a public health problem in Ethiopia. According to the World Health Organization, countries have to reduce undernutrition including child wasting to below 5% by 2025. Ethiopia is attempting to attain national and international targets of undernutrition while struggling with many problems.

**Objective:**

This study aimed to identify the prevalence and associated factors of wasting to provide information for further renewing policy commitments.

**Methods:**

We used community-based, cross-sectional data from the Ethiopian Mini Demographic and Health Survey. The survey was conducted in 9 regions and 2 city administrations. Two-stage cluster sampling was used to recruit study participants. In the first stage, enumerations areas were selected, and 28-35 households per enumeration area were selected in the second stage. Our analysis included 2016 women with children aged <5 years from the 2019 EMDHS data set. We dropped incomplete records and included all women who fulfilled the eligibility criteria. We used multilevel ordinal regression using Generalized Linear Latent and Mixed Models (GLLAMM) and predicted probability with log-likelihood ratio tests. Fulfilling the proportional odds model’s assumption during the application of multilevel ordinary logistic regression was a cumbersome task. GLLAMM enabled us to perform the multilevel proportional odds model using an alternative method.

**Results:**

In our analysis, wasting was 7.68% (95% CI 6.56%-8.93%). Around 26.82% of mothers never used antenatal care for their current child. Most mothers (52.2%) did not have formal education, and 86.8% did not have postnatal care for their children. Additionally, half (50.93%) of the mothers have ≥6 household members. Wasting was associated with feeding diverse foods (coefficient 4.90, 95% CI 4.90-4.98), female sex of the household head (–40.40, 95% CI –40.41 to –40.32), home delivery (–35.51, 95% CI –35.55 to –35.47), first (16.66, 95% CI, 16.60-16.72) and second (16.65, 95% CI 16.60-16.70) birth order, female child (–12.65, 95% CI –12.69 to –12.62), and household size of 1 to 3 (10.86, 95% CI 10.80-10.92).

**Conclusions:**

According to the target set by World Health Organization for reducing undernutrition in children aged <5 years to below 5% by 2025, child wasting of 7.68% in Ethiopia should spark an immediate reaction from the government and stakeholders. Informed policy decisions, technology-based child-feeding education, and food self-sufficiency support could improve the current challenges. Additional effort is important to improve low maternal education, family planning, awareness of sex preferences, women empowerment, and maternal health services.

## Introduction

### Background

Child wasting is a key indicator used by the World Health Organization (WHO) to estimate the prevalence of childhood malnutrition. Child wasting refers to children who are too thin for their height because of recent rapid weight loss or failure to gain weight. A wasted child is one whose weight falls below 2 SDs of what is expected for the child’s height. Moderately or severely wasted children are at a greater risk of death [1]. According to the United Nations Children’s Fund (UNICEF), child wasting due to acute undernutrition piles additional life-costing health risks on children and usually arises as the result of maternal undernutrition, low birth weight, poor feeding and care practices, infection, food insecurity, and poverty [2].

The short-term consequences of undernutrition include frequent sickness, delayed physical and mental development, restlessness, poor feeding, and low body weight. In long run, it causes stunting, inability to learn, poor health status, and poor work performance [3-5]. Unfortunately, childhood undernutrition constraints are disproportionally large in low-income countries [6]. Despite this, evidence indicates that the international community is fragmented in regard to engaging against the problem [7]. Currently, the WHO developed a strategic plan outlining the urgent and accelerated action to end hunger and all forms of undernutrition by 2030 [8]. The status of the plan was not assessed at present. As part of this initiative, the government of Ethiopia is committed to ending wasting and stunting in children aged ≤2 years by 2030, through the effective coordination and collaboration of sectors, communities, and development partners [9]. According to the Ethiopian Growth and Transformation Plan II (GTP-II) evaluation, the rate of wasting was 9% in 2015 and 4.9% in 2020 [10]. However, the Ethiopian Health Sector Transformation Plan endorsed in the same year as GTP-II showed that wasting was 7% in 2020 and should be 5% by 2025 [11]. The discrepancy encourages further investigation.

Specific observations show that undernutrition largely occurs during the early time of childhood when higher energy is necessary for growth and development [6,12]. In other words, UNICEF, WHO, and World Bank all agree that a child who experiences wasting has a reduced immunity against various childhood infections and possibly result in death or poor mental and physical development later [13]. Nutritional inadequacy during the first 2 years is also prevalent in some Asian countries [14]; however, the severity of the problem is higher in African countries due to additional access and awareness constraints [15].

A study conducted in 35 low- and middle-income countries showed that the rate of wasting was still 12.9% in these countries [16]. Stunting, underweight, and wasting combined in sub-Saharan countries ranged from 12.14% in Benin to 0.58% in the Gambia [17]. Recently, children in many African countries still experience undernutrition [18]. Some of the prevalence are 18% in Niger, 15.5% in Burkina Faso, 12.7% in Mali, 11.1% in Comoros, 8.7% in Ethiopia, 6.2% in Namibia, 13% in Chad, and 10.5% in Sao Tomé and Príncipe [19]. It is 5.3% in Ghana [20] and 25.5% in Baka Pygmy [21]. Wasting is common in children aged <5 years and school-age children in Ethiopia. A new small-scale study in Ethiopia showed that 15.3 % of children aged 6-14 years are wasted [22], whereas another study also showed that 6.3% of children aged 6-12 years in the South Gondar Zone are wasted [23]. Another study showed that among children aged 6-59 months, the rate of wasting was 16.8% in Kersa [24], 18.2% in Dabat district [25], and 12.8 % North Wollo [26], showing that there is high wasting in various corners of the country.

Recalling factors associated with wasting in low-income countries, one piece of evidence showed that child wasting was due to the quality of children’s diet, low birth weight, maternal undernutrition, and poor complementary feeding [15]. In Ghana, wasting was associated with the wealth index of households, maternal educational status, and region of residence [27]. Another study in South Africa stated that wasting was associated with food insecurity and low awareness [28]. Household wealth, iron deficiency, recent diarrhea, and exclusive breastfeeding were the main associated factors of wasting in Somalia [29]. Generally, the age of the child, sex of the child, birth order, size of the child, education of the mother, wealth index of the family, working status of the mother, antenatal care, and postnatal care for that child are factors associated with wasting in the sub-Saharan region [17].

In Ethiopia, one study showed that wasting was significantly associated with the types of toilets and the sex of the household head [30]. In another study, wasting was associated with poor dietary diversity, late initiation of breastfeeding, absence of postnatal vitamin A supplementation, and maternal occupational status [25]. Child age, sex, dietary diversity score, husband or partner’s educational level, and wealth index were associated with wasting in other studies [16,26,27,31-35]. According to the scaling-up management guideline, due to the climate liabilities and public health crises, Ethiopian children share a higher burden of wasting that needs undivided attention [36]. Despites this, relative to the WHO target for 2025, Ethiopia is struggling to reduce undernutrition of the targeted proportion [14]. Although wasting is an immediate, visible, and life-threatening form of undernutrition in children aged <5 years in peripheral and pastoralist areas in Ethiopia, studies usually do not focus on this undernutrition. Wasting is the first outcome of undernutrition, and early identification might help to control other undernutrition. It is challenging to comprehend wasting as a dichotomized variable because of its nature, which can be normal, moderate, or severe. Usually, studies dichotomize wasting as their main variable and this waste some information because of unnecessary merging. This has led to either over- or underestimation of wasting and not allowing appropriate policy decisions. Additionally, the inconsistent reports between GTP-II, Health Sector Transformation Plan, and some small scale studies mentioned above might show the existence of contextual gaps. The authors also believe that the unsuccessful previous efforts are because of the inability of identifying determinants in a country-representative sample that can show where to focus. Here, our main aim was to identify child wasting and its correlates using multilevel ordinary logistic regression to support policy direction.

### Conceptual Framework

**Figure 1:**
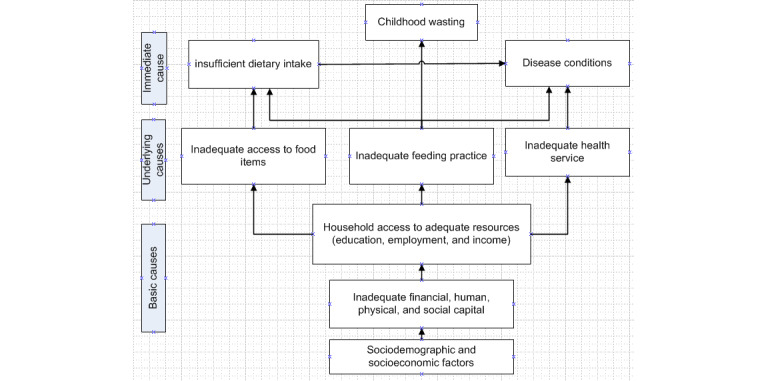
A conceptual framework adapted from United Nations Children's Fund (UNICEF) determinants for childhood wasting.

Figure 1 depicts a conceptual framework adapted from UNICEF’s conceptual framework for determinants of child wasting. It summarizes the sequence in which the child wasting occurs [37].

## Methods

### Data Sources and Participants

We used the 2019 Ethiopian Mini Demographic and Health Survey (EMDHS) data for this analysis. This data were collected for the second EMDHS in 2019. In Ethiopia, there are 4 administrative levels (Ethiopia or federal, regions, zones, and woredas). The 9 regions are further divided into city administrations (Addis Ababa and Dire Dawa), agrarian regions (Tigray; Amhara; Oromia; Benishangul-Gumuz; Southern Nations, Nationalities, and People’s Region; Gambela; and Harari), and pastoralists regions (Afar and Somali). We obtained data for 2016 eligible women with children aged <5 years from the 2019 EMDHS data set, downloaded from the MEASURE program web address, and extracted data elements necessary for this analysis. EMDHS 2019 used a sampling frame of all census enumeration areas (EAs) created for the 2019 Ethiopia Population and Housing Census (PHC) prepared by the Central Statistical Agency. There was a complete list of 149,093 EAs created for the 2019 PHC. An EA is a geographic area covering an average of 131 households. The sampling frame contained information about the EA location, type of residence (urban or rural), and an estimated number of residential houses focusing on key indicators for this survey. Each region was stratified into urban and rural areas, yielding 21 sampling strata. Samples of EAs were selected independently in each stratum in 2 stages. Finally, 305 EAs (93 in urban and 212 in rural areas) were selected with a probability proportional to EA size (based on the 2019 PHC frame) and with an independent selection in each sampling stratum. Either all women aged 15-49 years who were permanent residents of the selected households or visitors who slept the night before the survey were eligible for interview. The height and weight of children aged 0-59 months were collected, and women aged 15-49 years were interviewed using the Woman’s Questionnaire [37].

### Variables

The outcome variable of this study was wasting in children aged <5 years: if a child’s weight for height is below 2 SDs from the expected weight-for-height median identified by the WHO for boys and girls [26]. In this study, we classified children as normal (when the weight-for-height *z* score is between –2 SDs to 2 SDs) [28]; moderate wasting (when the weight-for-height *z* score is between –3 SDs to –2 SDs); and severe wasting (when the weight-for-height *z* score is below –3 SDs), depending on the references from WHO 2006 guideline [38].

### Independent Variables

We selected the following independent variables based on performance in previous evidence [17,23,25,26,30,32,33,39] and the availability of variables in the 2019 EMDHS data set. Age (mother and child), sex of the child, mother’s educational status, head of household, wealth index, religion, residence, antenatal care, place of delivery, postnatal care, breastfeeding, anemia status of the mother, anemia status of the child, dietary diversity score, husband or partner’s educational level, and birth order were independent variables.

### Variable Definitions

#### Antenatal Care

Antenatal care visits were presented in groups of none, 1-3, or 4+. A mother may have no visits, 1-3 visits, or over 4 visits according to WHO.

#### Anemia Status of the Mother

The anemia state of the mother was defined as the percentage of women aged 15-49 years with mild, moderate, or severe anemia or with any anemia. It is the number of not pregnant women whose hemoglobin count is less than 12.0 grams per deciliter (g/dL) plus the number of pregnant women whose count is less than 11.0 g/dL.

#### Anemia Status of the Child

The anemia state of the child was defined as the percentage of children aged 6-59 months with mild, moderate, or severe anemia or with any anemia. This is when the hemoglobin count of a child is less than 11 grams per deciliter (g/dL).

#### Dietary Diversity Score

The dietary diversity score measured children aged <5 years who consumed a minimum of 5 of the 8 food groups (grains, roots and tubers, legumes and nuts, dairy products, meat [fish, poultry, and liver/organ meats], eggs, vitamin A–rich fruits and vegetables, other fruits and vegetables, and breast milk) in the past 24 hours.

#### Household Head

Households were the primary unit selected for interview in the Ethiopian Demographic and Health Survey (DHS). The definition of a household is a person or group of related or unrelated persons who live together in the same dwelling unit(s), who acknowledge one male or female adult as the head of the household, who share the same housekeeping arrangements, and who are considered a single unit.

#### Family Wealth Index

The questionnaire included queries concerning the household’s ownership of several consumer items such as television and car; dwelling characteristics such as flooring material, type of drinking water source, toilet facilities; and other characteristics related to wealth status. Each household asset for which information was collected is assigned a weight or factor score generated through principal components analysis. The resulting asset scores were standardized about a standard normal distribution with a mean of 0 and an SD of 1. These standardized scores were then used to create the breakpoints that define wealth quintiles as lowest, second, middle, fourth, and highest [40].

### Data Processing and Analysis

We used frequencies, weighted frequencies, means, SDs, and percentages or proportions to describe child wasting. Our data set contained many factors, so we checked multi-colinearity using the mean variance inflation factor (1.31), which was within an acceptable range. Before applying different models for analysis, we cleaned the data per the study criteria in Stata software (version 15.0; StataCorp) and weighted considering sampling weight, primary sampling unit, and strata before analyzing. Since wasting has normal, moderate, and severe categories, we applied multilevel ordinary logistic regression to fit the data. We conducted a bivariate analysis to identify candidate variables for multivariate analysis and selected variables with a *P* value <.20 [40,41] for the multivariable model. In current statistical analyses, studies conduct preanalysis filters to select variables for final models at a *P* value <.25 [42] and commonly at *P*<.20 [43]. We declared the final association at a *P* value of <.05. We presented the outputs of the model using coefficients and 95% CI.

### Statistical Analysis—The Ordinal Logistic Regression Model

The data used in this analysis were hierarchical, which we could not analyze using binary logistic regression. Multilevel logistic regression was also not applicable since the response variable contained more than 2 categories. Therefore, we applied a special type of ordinal logistic regression (Generalized Linear Latent and Mixed Model [GLLAMM]) to account for the parallel planes and proportional odds assumptions. This model has been used to analyze clustered data [44]. We executed a mixed-effects ordinal logistic regression in a multilevel proportional odds model using GLLAMM. We used adaptive quadrature to estimate deviance and log-likelihood [45-47]. After fitting the full model, we also estimated posterior means and SDs of the latent variable. The marginal test gave us the expected response regarding the prior distribution of the latent variables so that we were able to look at the “marginal” or population-averaged effects of covariates [44,48].

### Ethical Considerations and Consent to Participate

This study used secondary data from demographic and health survey data files. Initially, the authors formally requested access to the data sets from the MEASURE DHS team by completing the web-based request form [49]. Accordingly, permission to access the data and the letter of authorization were obtained from ICF International. Therefore, for this study, consent to participate is not applicable. We kept all data confidential, and no effort was made to identify households or individuals. The Ethiopian Health Nutrition and Research Institute Review Board and the National Research Ethics Review Committee at the Ministry of Science and Technology of Ethiopia approved EMDHS 2019. The original informed consent allowed the free deidentified secondary analysis without additional consent. The authors also confirmed that all methods were carried out with relevant guidelines and regulations. The authors also ensured the study data were anonymous or deidentified for the confidentiality and privacy of the participants. According to the original consent, there was no compensation for this cross-sectional data acquisition.

## Results

We analyzed the data of 2016 mothers with children aged <5 years and found an overall wasting of 7.68% (95% CI 6.56%-8.93%). Using weighted frequencies, we found that 1.86% of children were severely wasted and 5.79% were moderately wasted. Around half (48.68%) of the mothers of the children were in the age range of 25-34 years, where 26.82% of them did not have antenatal care for the current child. Over half (52.2%) of the mothers had no education, and 86.8% never had postnatal care for their current child. Half (50.93%) of the mothers had ≥6 household members. Additionally, 24.93% of mothers had a history of having 6 or more childbirths. More exceptionally, 36.6% of mothers gave birth to 2 children in the last 5 years. Many mothers (39.85%) did not use iron supplementation in the previous pregnancy. Additionally, 37.49% of mothers did not give their children solid or semisolid food yesterday, whereas 22.9% did not provide diverse food for their children (Table 1).

In the GLLAMM, variables such as giving diverse food to a child, sex of the child, total household members, sex of household head, wealth index, birth order, and maternal education were significantly associated with child wasting (all *P*<.001). Accordingly, a child fed diverse food had 4.90 times higher log odds of having normal body weight when other factors were kept constant (coefficient 4.90, 95% CI 4.90-4.98). If the sex of the household head was female, the log odds of having normal body weight was 40.40 times lower (–40.40, 95% CI –40.41 to –40.32), and when the child delivered at home, the log odds of having normal body weight was 35.51 times lower (–35.51, 95% CI –35.55 to –35.47). In other words, first and second birth-order children had higher log odds of falling into the normal body weight category (16.66, 95% CI 16.60-16.72; and 16.65, 95% CI 16.60-16.70, respectively). Families in the poor or middle-income category had 5.95 and 5.94 times lower log odds of having normal body weight (–5.95, 95% CI –6 to –5.90; and –5.94, 95% CI –6 to –5.88, respectively). The female sex of the child correlated with 12.65 times lower log odds of having normal body weight (–12.65, 95% CI –12.69 to –12.62) when all factors were constant. Children from smaller household sizes (1-3 and 4-5 members) had higher log odds of having the normal body weight (10.86, 95% CI 10.80-10.92; and 10.87, 95% CI10.91-10.92, respectively), and children whose mothers were educated (primary, secondary, and higher education) had higher log odds of having normal body weight (1.96, 95% CI 1.89-2.03; 1.99, 95% CI 1.92-2.04; and 1.99, 95% CI 1.90-2.10, respectively) (Table 2).

The equality between the nonproportional odds log-likelihood and postestimation log-likelihood shows that the assumptions hold for the model, and we applied the nonproportional model, although the proportional model almost performed similarly (Table 3).

**Table 1:** Sociodemographic characteristics of mothers in Ethiopia, from the 2019 Ethiopian Mini Demographic and Health Survey.

Variables		Weighted frequency, n (%)
**Educational level**		
	No education	1052.89 (52.2)
	Primary	745.1 (36.94)
	Secondary	386.69 (7.85)
	Higher	60.52 (3)
**Number of children**		
	0-1	1113.56 (55.21)
	2-4	738.13 (36.6)
	≥5	165.26 (8.19)
**Timing of the initial ANC^a^**		
	First trimester	541.28 (36.67)
	Second trimester	778.08 (52.71)
	Third trimester	156.68 (10.62)
**Total number of children ever born**		
	<6	1514.12 (75.07)
	≥6	502.81 (24.93)
**Wasting**		
	Severe	37.41 (1.86)
	Moderate	116.74 (5.79)
	Normal	1862 (92.36)
**Age group (years)**		
	15-24	548.74 (27.21)
	25-34	981.94 (48.68)
	35-49	486.25 (24.11)
**Number of times child was fed solid food**		
	0 (not fed)	756.05 (37.49)
	1-2	431.74 (21.41)
	3-4	604.55 (29.97)
	≥5	224.58 (11.13)
**Wealth status**		
	Poor	896.88 (44.47)
	Middle	348.89 (17.3)
	Rich	771.15 (38.23)
**Feeding diverse food**		
	No	461.87 (22.9)
	Yes	1555.06 (77.1)
**Took iron during ANC**		
	No	803.73 (39.85)
	Yes	1213.19 (60.15)
**Number of household members**		
	1-3	266.84 (13.23)
	4-5	722.9 (35.84)
	≥6	1027.19 (50.93)
**Number of ANC visits**		
	None	540.88 (26.82)
	1-2	239 (11.86)
	3-4	808.95 (40.11)
	≥5	427.92 (21.22)

^a^ANC: antenatal care.

**Table 2:** The Generalized Linear Latent and Mixed Models of wasting in children aged <5 years in Ethiopia, from the 2019 Ethiopian Mini Demographic and Health Survey.

Variables			Coefficient (SE)		95% CI	*P* value	
**Diverse food fed to child**							
	No	—^a^		—			
	Yes	4.9 (.02)		4.90 to 4.98		.001	
**Give milk to child**							
	No	–9 (.024)		–9.04 to –8.96		.001	
	Yes	—		—			
**Sex of household head**							
	Male	—		—			
	Female	–40.4 (.022)		–40.41 to –40.32		.001	
**Birth order**							
	First	16.66 (.03)		16.60 to 16.72		.001	
	Second	16.65 (.026)		16.60 to 16.70		.001	
	Third or above	—		—			
**Place of delivery**							
	Home	–35.51 (.026)		–35.55 to –35.47		.001	
	Health facility	—		—			
**Wealth index**							
	Poor	–5.95 (.02)		–6 to –5.90		.001	
	Middle	–5.94 (.30)		–6 to 5.88		.001	
	Rich	—		—			
**Sex of the child**							
	Male	—		—			
	Female	–12.65 (.017)		–12.69 to –12.62		.001	
**Number of household members**							
	1-3	10.86 (.03)		10.80 to 10.92		.001	
	4-5	10.87 (.02)		10.91 to 10.92		.001	
	≥6	—		—			
**Maternal education**							
	No education	—		—		.	
	Primary	1.96 (.04)		1.89 to 2.03		.001	
	Secondary	1.99 (.03)		1.92 to 2.04		.001	
	Higher	1.99 (.48)		1.90 to 2.10		.001	

^a^Not applicable.

**Table 3:** Parameter estimates and tests for wasting in children aged <5 years in Ethiopia, from the 2019 Ethiopian Mini Demographic and Health Survey.

Parameters	Proportional odds model	Nonproportional model
Variance	1.59	1.50
SE	.37	.36
Intracluster correlation coefficient	.32	.32
Log-likelihood of the model	–666	–671
Akaike information criteria of the model	570	558
Bayesian information criteria of the model	637	635
Severe stunting, σ (SE)	–4.44 (.74)	–3.84 (.51)
Moderate stunting, σ (SE)	–2.90 (.73)	–2.32 (.50)
Proportional model nested in nonproportional model	Likelihood ratio test: *χ*^2^_9_=11.04, *P*=.002	—^a^
Predicted probability or marginal test	Log-likelihood	–671

^a^Not applicable.

## Discussion

### Principal Findings

From our analysis, the overall wasting among children aged <5 years was 7.68% (95% CI 6.56%-8.93%). This finding is consistent with the 7.4% and 9.5% wasting rates in Afghanistan and India, respectively [27]. The finding is less than the average wasting rate (12.14%) in sub-Saharan countries [17]. The rate was 18% in Niger, 15.5% in Burkina Faso, 12.7% in Mali, and 11.1% in Comoros [19]. It is less than other study findings in Ethiopia that show wasting was 15.3% in Northwest Ethiopia [22], 16.8% in Kersa [24], 18.2% in Dabat district [25], and 12.8 % in North Wollo [26]. The clear discrepancy might show that Ethiopia needs to work very hard to reach the less than 5% wasting target set by WHO for 2025 [14]. Additionally, 52.2% of the mothers did not have an education. This is also evidenced by the 60.25% of those with no educational attainment from country-level studies in 2019 [39]. From other evidence, 59.7% of mothers have no education in the country [50], and other studies have confirmed the same [33,51,52]. Education is repeatedly the factor behind the most underachievement in the country and needs further efforts. Nearly forty percent (39.85%) of mothers did not use iron supplementation during pregnancy, which is also consistent with a finding of 34.6% in Somali [29] but sufficiently different from prenatal iron use of 45.3% in a community-based study in the country [22]. Half (50.93%) of mothers have 6 or more children, which is greater than the finding of 30.8% from another study [53]. A poor wealth index of 44.47% is consistent with the finding of 44.2% in another study [54] that shows birth control and economically empowering mothers could be the crucial target for future improvement.

There are different factors associated with all underachievements. Children who were fed diverse foods by their mothers had higher log odds of normal body weight. The finding is consistent with the results in other studies in the country [26,51]. Ethiopia has diverse cultures, which are the base for various feeding styles; however, as things change over time, natural products are no longer available in sufficient volumes and mothers might need further health education [55,56]. Female household heads were associated with the poor body weight of their children aged <5 years. In Ethiopia, men lead the household and assume all responsibilities [57]. However, when the family is separated or divorced, finding food and feeding children lies with women [37]. This might result in the potential for the undernutrition of children [39,58]. Women are poor and undereducated and have low awareness in the country, which needs attention when household responsibility falls into their hands [37,58]. Home delivery is associated with poor feeding practices (wasting) in children aged <5 years, as was also observed in other studies [31,33]. The reason might be the exposure of mothers to health professionals’ counseling, which could be useful for the future. However, mothers who gave birth in health institutions might be mothers who already know what to do. Therefore, careful intervention selection is necessary. Additionally, children with first or second birth order might get enough attention during development, as opposed to those born later. This information is also available from studies conducted in various parts of the country [20,39]. A planned and economically sized family is always important. It seems that those born with many children do not give equal attention to every child, which needs attention [50]. Consequently, wasting was largely experienced by large-family children. This is consistent with another study conducted in the country [31]. The essence of family planning to reduce the risk of big families and maintain already large families might be vital. Moreover, female children had higher log odds for moderate or severe wasting, which is also consistent with another study [33]. Thus, the problem of family planning and management might have reduced most of these feeding problems. In most studies in the country, maternal education is the most prevalent problem, which was also seen in this study [33,49,51,59-61]. Generally, the current problems could have been reduced by maternal education, family planning, avoidance of sex preference, and access to pregnant women’s services. Despite all the essential findings of this study, there are also some possible limitations. Disproportion of sampling, missing data, and secondary nature of the data are some limitations. The authors approached the problem through multilevel analysis, weighting, dropping records with missing data, and considering the time of data collection in the discussion.

### Conclusions

Considering the target set by WHO to reduce undernutrition in children aged <5 years to below 5% by 2025, the current 7.68% wasting rate in children aged <5 years is very high and should spark immediate commitment from the government. Additionally, improving low maternal education, supporting women to use family planning, creating awareness of sex preferences, empowering women economically, and renewing commitment to improving maternal health services and follow-ups during pregnancy might be inclusive activities that need further encouragement. It was suggested that revisiting policy with current technology-based child-feeding education and advocating food self-sufficiency in the country could improve the current challenges

## Abbreviations

DHS: Ethiopian Demographic and Health Survey
EA: enumeration area
EMDHS: Ethiopian Mini Demographic and Health Survey
EPHC: Ethiopia Population and Housing Census
GLLAMM: Generalized Linear Latent and Mixed Models
GTP-II: Growth and Transformation Plan II
UNICEF: United Nations Children’s Fund
WHO: World Health Organization

## Data Availability

The survey data set used in this analysis is third-party data from the Demographic and Health Survey (DHS) website (www.dhsprogram.com) and permission to access the data is granted only to the registered DHS data users.
